# Effect of Handrail Height on Sit-To-Stand Movement

**DOI:** 10.1371/journal.pone.0133747

**Published:** 2015-07-24

**Authors:** Satomi Kinoshita, Ryoji Kiyama, Yoichi Yoshimoto

**Affiliations:** 1 Doctor Course, Graduate School of Health Sciences, Kagoshima University, Kagoshima, Japan; 2 Course of Physical Therapy, School of Health Sciences, Faculty of Medicine, Kagoshima University, Kagoshima, Japan; Tokai University, JAPAN

## Abstract

**Background:**

Care-needing older adults and disabled individuals often require handrails for assistance of movements, such as sit-to-stand movements. Handrails must be set at the appropriate position; however, the effects of handrail height on joint movement and center-of-gravity movements during sit-to-stand movement remain unclear. In the present study, we sought to clarify the effects of handrail height on joint movement, center-of-gravity, and floor reaction force during sit-to-stand movement.

**Methods:**

Subjects included 16 healthy young adults and 25 older adults who require long-term care. Kinetic and kinematic measurements during sit-to-stand movement of young adults were conducted using a 3-D motion analyzer and a force plate. Trunk forward tilt angle during sit-to-stand movement of older adults was measured using a still image from a video recording.

**Results:**

Using low handrails, sit-to-stand movement resulted in an increased hip flexion angle, ankle dorsiflexion angle, and trunk forward tilt angle and a greater forward center-of-gravity shift than when not using handrails in young adults during seat-off. In contrast, using high handrails resulted in a smaller hip flexion angle and trunk forward tilt angle in young adults. The backward force on the floor was decreased in the low handrail condition, and was increased in the high handrail condition rather than that of sit-to-stand movement without handrails in young adults. The effect of handrail height on trunk forward tilt angle was the same in both healthy young adults and care-needing older adults during seat-off.

**Conclusion:**

Because handrail height affects joint movement and shift in the center-of-gravity during sit-to-stand movement, handrail position should be selected to match the status of older adults with functional impairment.

## Introduction

Handrails are used by care-needing older adults and disabled individuals to stabilize their movements during many daily situations. Most studies on handrails have examined walking movement [1–4]. Use of handrails reportedly contributes to an increase in walking speed, reduction in pain while walking, reduction in energy cost, and increase in gait balance control [1–3]. It has additionally been reported to aid in fall prevention [5]. However, handrails are also frequently used in situations other than walking, such as sit-to-stand (STS) movement. When rising from a chair, the base of support narrows from a previously wide, stable seated position, and the center of gravity (COG) shifts forward. This shift in the COG can greatly throw off one’s balance. Accordingly, numerous studies have been conducted on STS movement looking at kinematic characteristics and diseases as well as changes in characteristics of kinematic disorders with aging [6–12]. Fall prevention is critical for care-needing older adults, in whom falls can result in bone fractures, becoming bedridden, need for long-term care (LTC), and even death. The decrease in physical functioning with aging in care-needing older adults has been shown to result in reduced motor control ability and the external environment exerting a greater impact on STS movement [13–15]. A few studies have analyzed the relationship between handrail position as an environmental factor and STS movement from a chair [16].

Important elements in natural rising from a seated position are the forward shift of the trunk, the shift of the COG to a base of support comprised of the feet, the lifting of the gluteal region from the seat, and the upward shift of the COG due to extension of the knee and hip joints [17–19]. The handrail must be set at an appropriate position to assist the difficult phase of the user during STS movement. In a previous study, we examined the effects of handrail height on lower limb joint moment and joint load during STS movement [20]. We found that high handrails decreased the total lower limb moment more than low handrails, and that use of high handrails reduced hip joint load by 55% compared to STS movement without any handrails. Factors involved may be changes in joint movement with handrail height and resulting changes in the shift of the COG and floor reaction force (FRF). However, the effects of handrail height on joint movement, COG, and FRF movements during STS movement remain unclear.

We therefore set out to clarify the effects of handrail height on joint movement, COG, and FRF during STS movement. We mainly examined kinematic elements in healthy young adults. However, major users of handrails are care-needing older adults. As a previous study showed that the movement pattern during STS differs between healthy young adults and care-needing older adults [21], we examined the latter as well.

## Methods

Subjects were 16 healthy young adults aged 21–43 years (15 men and one woman; mean age, 23.2 ± 5.3 years; mean height, 172.6 ± 5.8 cm; mean weight, 65.2 ± 6.7 kg; mean ± standard deviation) and 25 care-needing older adults aged 65–93 years (three men and 22 women; mean age, 77.5 ± 8.4 years; mean height, 152.3 ± 8.0 cm; mean weight, 51.6 ± 7.3 kg). Healthy young adults were excluded if they had a history of a musculoskeletal or neurological disorder. Care-needing older adults included two people with cancer, six with Parkinson’s syndrome, five with diabetes mellitus, five with cerebrovascular diseases, two with cardiovascular diseases, two with respiratory diseases, one with visual impairment, and two with arteriosclerosis obliterans. Care-needing older adults used a handrail during STS movement as usual, and were excluded if they had dementia. All were physically capable of performing STS movement independently using a handrail. Comparison of the characteristics in the two groups revealed significant differences in height and weight (P < .001 for both; paired t-test). Written informed consent was obtained from all participants after explanation of the details of this study. The study protocol was approved by the Clinical Review Board of the Faculty of Medicine, Kagoshima University.

A 3-D motion analyzer (VICON MX3; Oxford Metrics, Oxford, UK) comprising seven cameras and three force plates (OR6-7, BP400600; Advanced Mechanical Technology, MA, USA) were used to perform kinetic and kinematic measurements during STS movement by healthy young adults. We used three force plates, two of which were placed below the right and left feet respectively, and the third of which was placed under the chair. The sampling frequency was 100 Hz for the motion data and 1000 Hz for the FRF. A video camera (Sony DCR150; Sony Inc., Tokyo, Japan) was used to perform kinematic measurements on care-needing older adults, with a sampling frequency of 60 Hz.

STS movement was compared for three conditions: no handrail (“Without”), high handrails (“High”), and low handrails (“Low”). Height-adjustable handrails were placed on either side of the participant. The subjects were instructed to grip the handrail in a way that facilitated the STS movement and to perform the STS movement at a spontaneous speed. Both arms were crossed above the chest during the Without condition.

**Fig 1:**
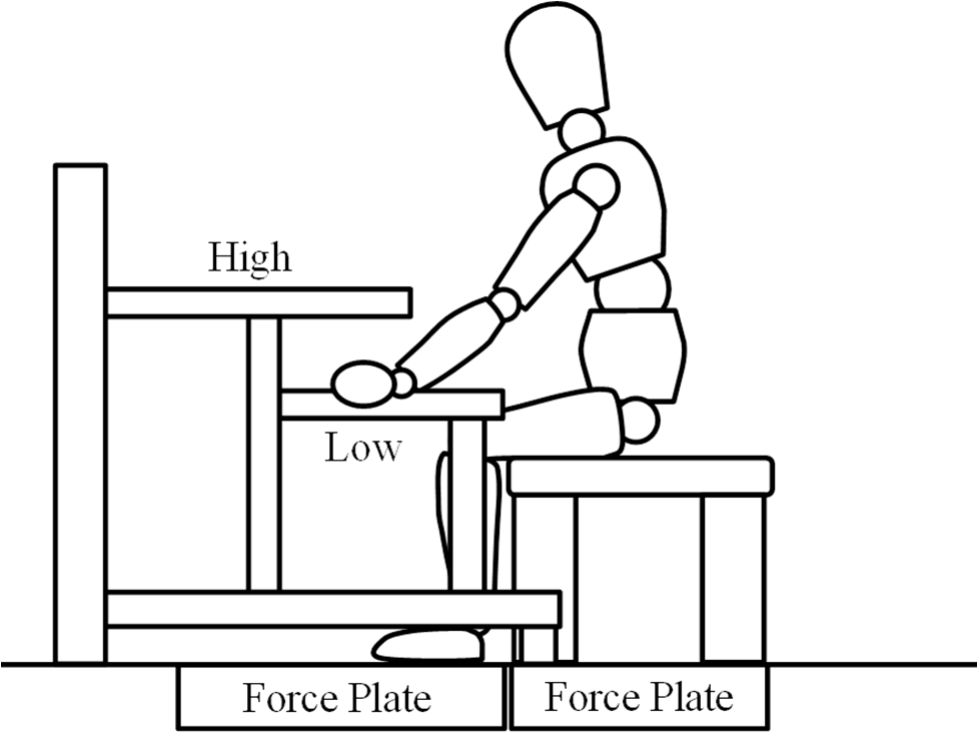
Schematic drawing of the motion analysis for young adults during STS movement with and without handrail support. The FRF of the bilateral lower extremity and chair were measured using three force plates, respectively. The height of the handrails was adjusted to the greater trochanter in the standing position (High) or the sitting position (Low), bilaterally.

The height of the handrails was adjusted to the greater trochanter in the standing position (High) or the sitting position (Low), bilaterally. At the start, the participant was seated with their knee at 90 degrees degrees (Fig 1). The order of conditions was randomized and measurements were taken after three practice trials. The average for the bilateral lower limb in three measurements was used for data analysis.

Reflective markers were attached to landmarks on the healthy young adults prior to taking measurements. The Plug-in-Gait model was used for the marker set and analyzed with the 3-D motion analyzer and force plate [22]. For care-needing older adults, reflective markers were attached to the acromion, greater trochanter, lateral epicondyle of the femur, and ankle joint lateral malleolus.

Seat-off is a key instance in STS movement when postural control is most difficult and there is a large load on the lower limb, and has been analyzed by Mark et al [11] and Papa et al [23]. The seat-off event was decided by the FRF of the chair for young adults, and by visual observation for care-needing older adults. Therefore, we analyzed the kinetic and kinematic data at seat-off. Segments were defined and the forward tilt angle of the trunk, hip flexion angle, knee flexion angle, and ankle dorsiflexion angle when the gluteal region left the chair (“seat-off”), and COG during seat-off were measured on the sagittal plane. The forward tilt angle of the trunk was calculated as the angle between the trunk segment and vertical line; a positive value indicates the forward tilt. The vertical and forward/backward components of the FRF were normalized according to the participant’s weight. The **instance** position of the COG was calculated as the position relative to the ankle joint; positive values indicate a forward shift of the COG. ImageJ (National Institutes of Health; Bethesda, MD, USA) was used to analyze data from care-needing older adults. Positional information from the acromion and greater trochanter markers was used to calculate the trunk forward tilt angle at seat-off. Prior to this study, we tested the consistency between the trunk tilt angles during STS movement measured by the 3-D motion analysis and the video camera using the intraclass correlation coefficient (ICC). A high consistency was observed between those angles (ICC_(2,1)_, 0.971; P < .001).

One-way repeated measures analysis of variance (ANOVA) was performed with handrail height as a factor to test the effects of the handrail on the various indicators in healthy young adults. Two-way repeated measures ANOVA was performed to test the trunk forward tilt angle at seat-off with group (healthy young adults vs. care-needing older adults) and handrail height as factors. Prior to the repeated measures ANOVA, we tested the sphericity. If sphericity was not assumed, the Greenhouse–Geisser method was used for analysis. When a significant interaction was found, an unpaired t-test was used to examine the differences between groups, and one-way ANOVA was used to examine the effects of handrail height. The Bonferroni method was used for the post hoc test. All statistical analyses were performed with SPSS 20.0 (SPSS; Chicago, IL, USA), with a level of statistical significance of 5%.

## Results

For healthy young adults, a significant difference was observed between all conditions for hip (Table 1; Figure A in S1 Fig; F_(2,30)_ = 36.3, P < .001) and knee (Figure B in S1 Fig; F_(2,30)_ = 6.0, P = .006) flexion angle and ankle dorsiflexion angle (Figure C in S1 Fig; F_(2,30)_ = 5.7, P = .008). The hip flexion angle was significantly larger in the Low than in the Without condition (P = .006). In contrast, the hip flexion angle was smaller in the High than in the Without condition (P = .005). Although the knee flexion angle was larger in the Low than in the Without condition (P = .003), this difference was negligible (2 degrees). The ankle dorsiflexion angle was significantly larger in the Low than in the Without condition (P = .004). The COG in the Low condition was located more anterior than that in the other two conditions during seat-off (S2 Fig; F_(1.4,20.4)_ = 24.4, P < .001).

**Table 1:** Results of the joint angle, shift of the COG, and FRF in young adults.

	Hip flexion angle (deg)	Knee flexion angle (deg)	Ankle dorsiflexion angle (deg)	Forward COG shift (mm)	Backward force of FRF (N/kg)	Vertical force of FRF (N/kg)
Without	93.3 ± 9.0** ††	82.7 ± 7.4**	9.0 ± 4.6**	18.2 ± 30.6**	0.84 ± 0.31**	6.1 ± 0.8** ††
Low	99.4 ± 8.5††	84.7 ± 7.4	11.5 ± 4.8	52.1 ± 31.0††	0.56 ± 0.28††	5.2 ± 0.8††
High	87.6 ± 8.1	84.3 ± 7.5	10.0 ± 5.3	25.5 ± 34.3	0.89 ± 0.28	5.7 ± 0.9

These values are data at seat-off moment. The forward shift of the COG was calculated as positions relative to the ankle joint. The mean and standard deviation are shown.

Note. COG, Center of Gravity; FRF, Floor Reaction Force.

**P < .01 significant difference between Low condition.

††P < .01 significant difference between High condition.

The backward FRF differed significantly with handrail height, being highest in the High condition and lowest in the Low condition (Figure A in S3 Fig; F_(2,30)_ = 33.4, P < .001). The vertical FRF differed significantly with handrail height, being significantly lower in the Low and High conditions compared to the Without condition (Figure B in S3 Fig; F_(2,30)_ = 70.1, P < .001).

**Fig 2:**
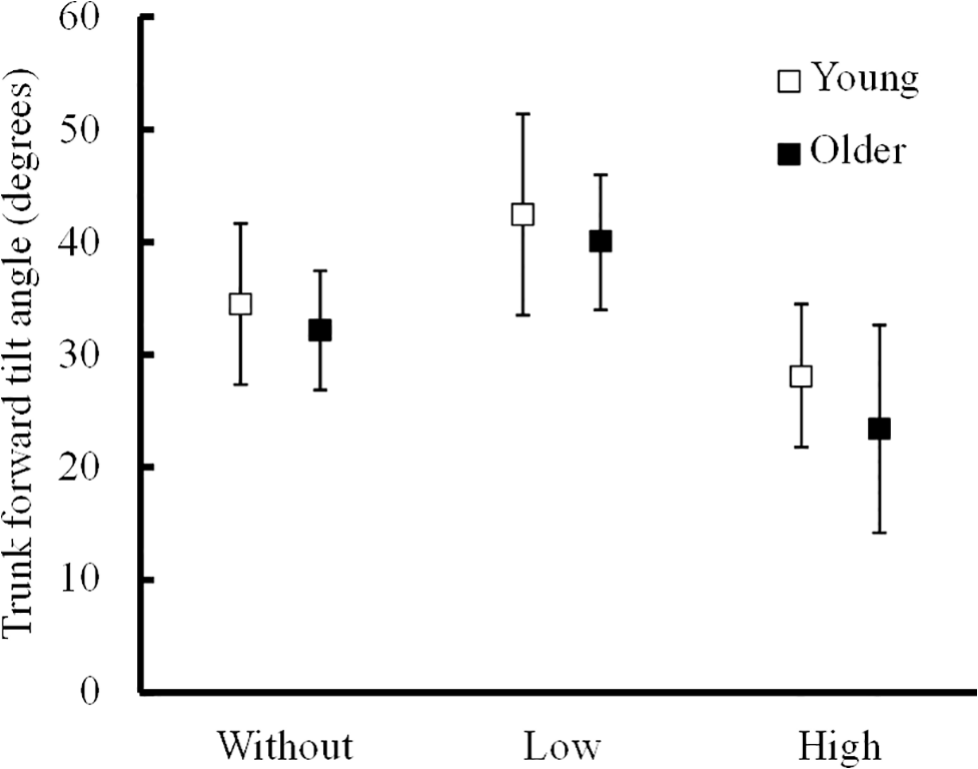
Average and standard deviation of forward tilt angle of trunk in young adults and care-needing older adults during STS movement. The handrail position had similar effect on the forward tilt angle of trunk for the two groups, showing no significant difference.

A significant interaction was seen in the trunk forward tilt angle between healthy young adults and care-needing older adults (Fig 2; F_(2,78)_ = 5.2, P = .008). For care-needing older adults, trunk forward tilt angle was 32.2 ± 5.3° in the Without condition, 42.4 ± 8.9° in the Low condition, and 23.4 ± 9.2° in the High condition. This angle differed significantly between all handrail conditions for both healthy young adults (Figure D in S1 Fig; F_(2,30)_ = 37.8, P < .001) and care-needing older adults (F_(2,48)_ = 81.4, P < .001). The trunk forward tilt angle was significantly larger in the Low condition for both care-needing older adults and healthy young adults than in the other two conditions (P < .009). The angle did not differ significantly between healthy young adults and care-needing older adults in any condition (P > .106).

## Discussion

We tested the effects of handrail position on joint movement and COG shift during STS movement in healthy young adults and in care-needing older adults who actually require handrails. The vertical FRF during STS movement decreased significantly with both types of handrails, and the decrease was by 13.7% for the low handrail and 7.1% for the high handrail. We found that STS movement in the Low condition involved an increased hip flexion angle, ankle dorsiflexion angle, and trunk forward tilt angle and a greater forward COG shift than STS movement in the Without condition. In contrast, STS movement in the High condition involved a smaller hip flexion angle and trunk forward tilt angle than STS movement in the Without condition. The effect of handrail height on trunk forward tilt angle was the same in both healthy young adults and care-needing older adults.

Normal STS movement begins with a tilting forward of the trunk accompanying hip joint flexion and ankle dorsiflexion that shift the COG forward. The trunk forward tilt angle facilitates the shift of the COG to the base of support. Next, when the gluteal region lifts off from the chair, the base of support that was initially large, including the gluteal region, shifts to a narrow region comprised of the feet touching the ground, and the COG moves to the edge of the base of support, so that such seat-off motion brings the highest risk of falling. Furthermore, the FRF is the largest during this phase and the force is in the backward/upward direction. The hip joint and knee joint exert the maximum extension moment in response to this FRF [24]. This is the actual moment of seat-off and is a key phase in STS movement as the heavy load on the lower limbs makes postural control difficult.

When using low handrails, it is necessary to shift the COG forward for loading on the handrail. This may have prompted the flexion of the hip and forward bending of the trunk. Pushing on the handrail gives the user an upward/backward force from the handrail that may reduce the vertical and backward FRF. Therefore, low handrails may be effective for individuals whose COG does not shift forward sufficiently by trunk tilt during seat-off.

However, the increase in trunk forward tilt and backward FRF reduced during STS movement brings the FRF and the center of the knee joint closer together and increases the distance from the center of the hip joint, thereby reducing the load on the extensor muscles in the knee joints and increasing the load on the extensor muscles in the hip. It is therefore important to consider hip joint range of motion for flexion and extensor muscle strength for use of low handrails. Findings from our previous study are congruent with those of the present study, showing that centripetal power of the hip joints is larger when using low handrails than when not using any handrails [20].

STS movement with high handrails was associated with a decrease in hip flexion angle and trunk forward tilt angle and an increase in backward FRF than in STS movement without handrails. When pulling on handrails located in a high position, an upward/forward force may act on the body. Moreover, the backward FRF during seat-off was larger than regular STS movement, which may reflect a forward force from the handrail [16]. The forward and upward reactive force created from the handrail decreases the forward tilt of the trunk. This smaller trunk forward tilt angle and increased backward FRF then brings the FRF closer to the center of the hip joint and further from the center of the knee joint compared to the Without condition. This may then result in a decrease in the load to the hip extensor muscles and increase in the load to the knee extensor muscles. In addition, pulling on the handrail gives a forward/upward force on the body and reduces the propensity to fall backward. During this STS movement, arm compensation for the forward/upward shift in the COG due to the position of the handrail can enable assistance of the lifting of the COG that can potentially reduce the load to the hip extensor muscle in individuals with lower limb paralysis. This method of pulling on a handrail during STS movement involves a compensation of the COG shift with the arms that may result in a tendency for a stronger braking force in the lower limbs. As the reduced trunk tilt induced by the pulling force and increased backward FRF is different from the kinesiology of the natural STS movement, it is unlikely that STS with a high handrail would be linked to learning of the STS movement.

Aging is accompanied by a decrease in physical functioning, including reduced muscle strength in the lower limbs and trunk and limiting of the range of motion, as well as decreased functioning of the nervous system. These effects may have an impact on various elements of physical functioning. Age-related changes in physical condition have been widely reported to increase the difficulty of STS movement [19, 25–28]. An understanding of how to adjust the environment to enable easy and natural STS by older adults is critical [29–35]. Individuals differ in their degree and type of LTC needs; however, it is important to adjust the height of handrails to eliminate difficulty in performing STS movement due to physical changes such as reduced lower limb muscle strength and limited range of motion. Enhancing the STS movement environment may help raise the level of independence of care-needing older adults, and decrease assistance need and manpower cost. Achieving the best handrail position may be beneficial to both the care receiver and the caregiver. Adjustments to the living environment based on the individual’s level of physical functioning should include consideration of the positioning of handrails to assist in raising the gluteal region.

The effect of handrail height on trunk forward tilt angle was similar in healthy young adults and care-needing older adults. The positional relationship between the body and the handrail determines the mechanical effects that the handrail will have on the body. This may be why similar effects were observed in healthy young adults and care-needing older adults. It is therefore reasonable to consider the present findings applicable to care-needing older adults.

In the present study, we did not analyze the types of reactive forces from the handrails. Differences in characteristics between the groups, including height, weight, and gender distribution, may influence the STS movement. Moreover, only a kinematic analysis was performed on care-needing older adults. Further studies are needed to perform a thorough kinetic analysis, including the reactive forces from the handrail. Another limitation is that STS movement has been considered asymmetrical [36, 37]. Effects of leg dominance may therefore play a role. Neither dominant leg nor dominant hand was included in the criteria tested in the present study. Further tests that factor in hand and foot dominance are also needed.

## Conclusion

We hereby demonstrated that handrail height affects joint movement and shift in the COG during STS movement. The results showed that low handrails are preferable for individuals that are capable of tilting their trunk forward and have strong muscles around their hip. In contrast, high handrails were more useful for those with difficulty tilting their trunk forward and weak muscles around their hip. Handrail position should be selected accordingly to match the situation for older adults, among whom there is great variation in functional impairment.

## Supporting Information

S1 Fig The joint angle of hip (Figure A), knee (Figure B), ankle (Figure C) and trunk forward tilt angle (Figure D) during STS movement in a young adult. Vertical solid line indicates the seat-off.
(TIF)

S2 Fig The forward shift of the COG during STS movement in a young adult. The position of the COG was calculated as positions relative to the ankle joint. Vertical solid line indicates the seat-off.
(TIF)

S3 Fig The backward force (Figure A) and the vertical force (Figure B) of FRF during STS movement in a young adult. Vertical solid line indicates the seat-off.
(TIF)
