# Supplementary figures and images for: Effect of Handrail Height on Sit-To-Stand Movement
Source: PLoS One. 2015 Jul 24;10(7):e0133747. doi: 10.1371/journal.pone.0133747 (PMC4514829; doi:10.1371/journal.pone.0133747)

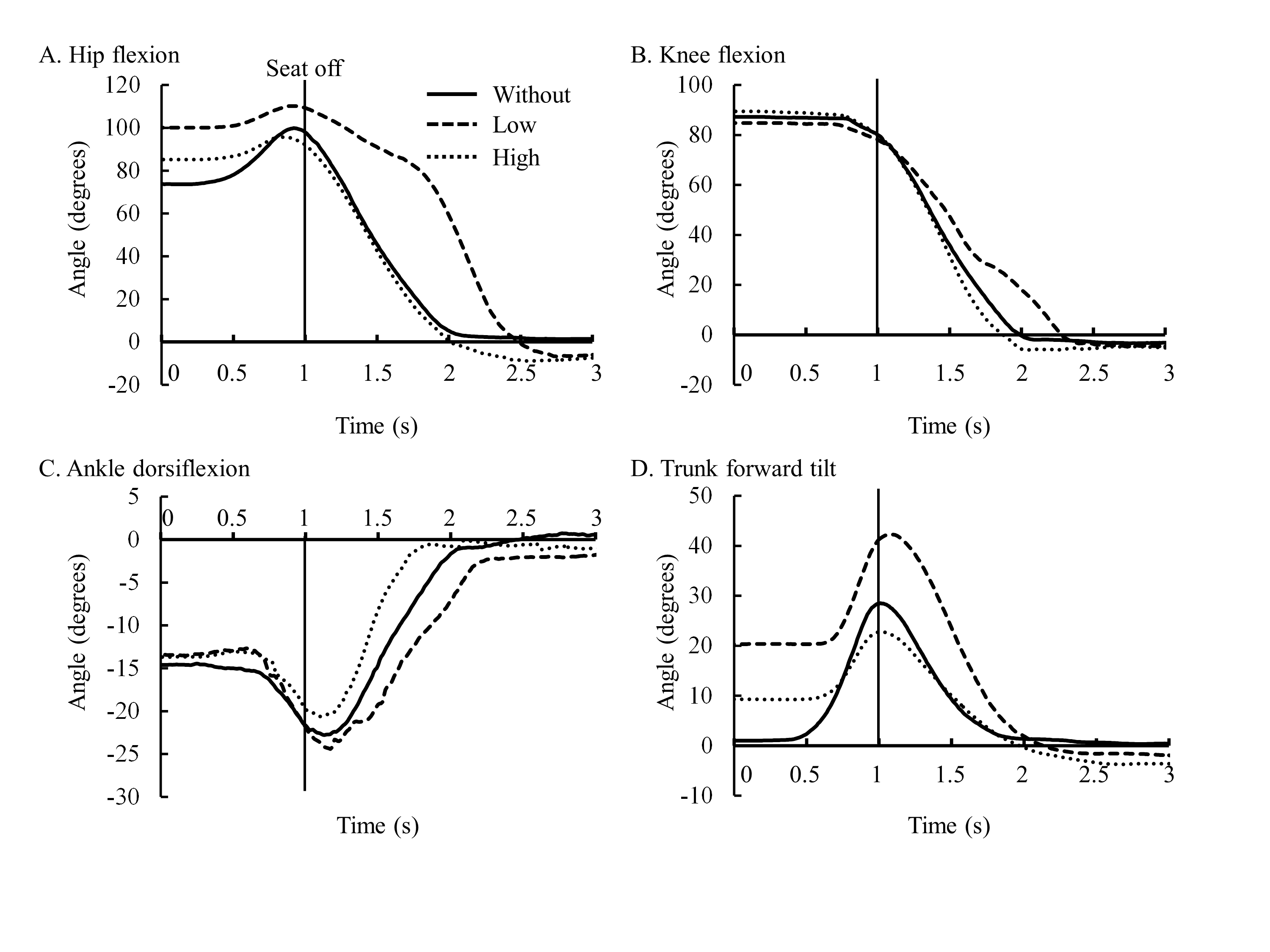

Supplement: S1 Fig — Vertical solid line indicates the seat-off. (TIF) [file pone.0133747.s001.tif]

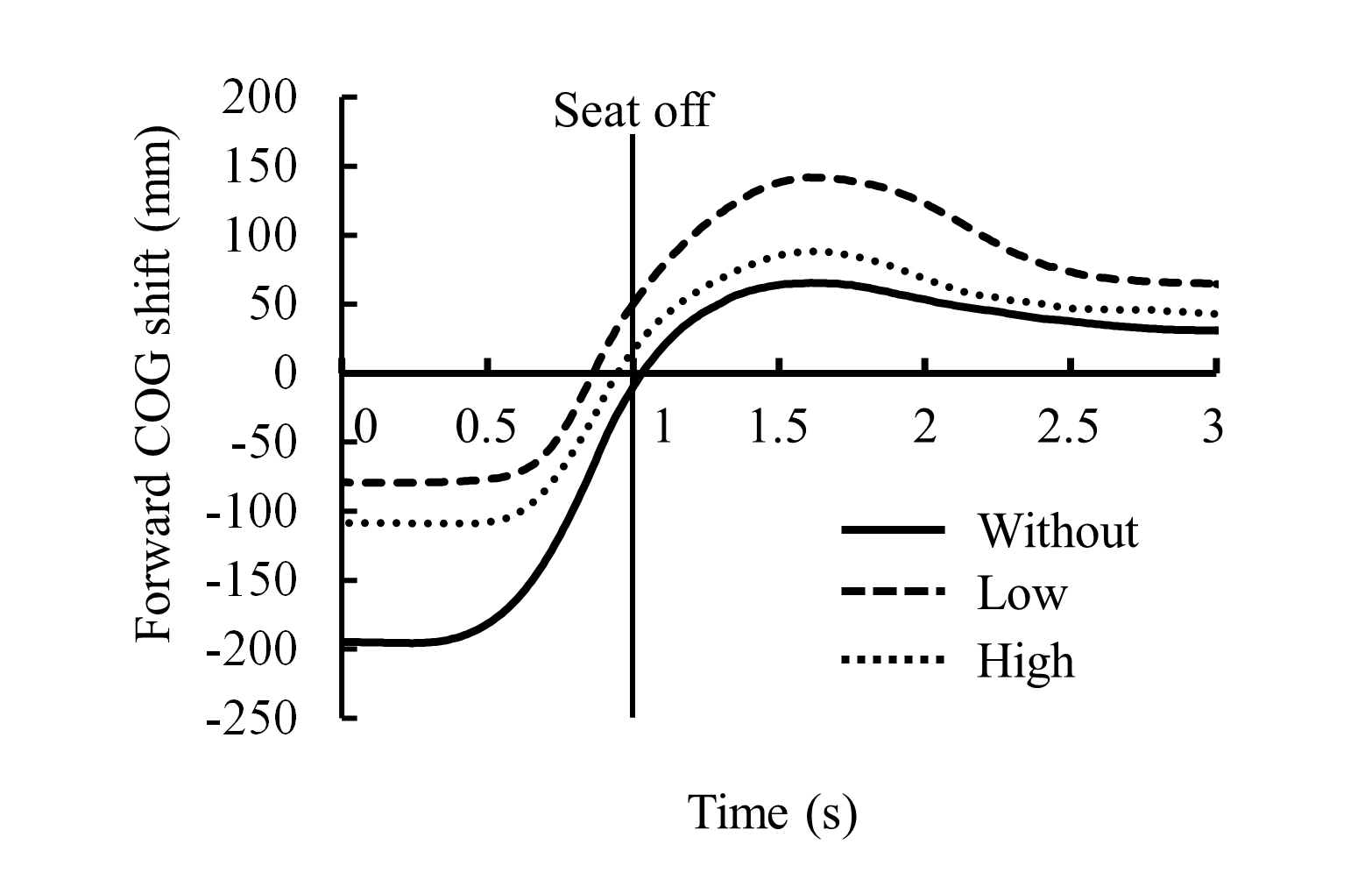

Supplement: S2 Fig — The position of the COG was calculated as positions relative to the ankle joint. Vertical solid line indicates the seat-off. (TIF) [file pone.0133747.s002.tif]

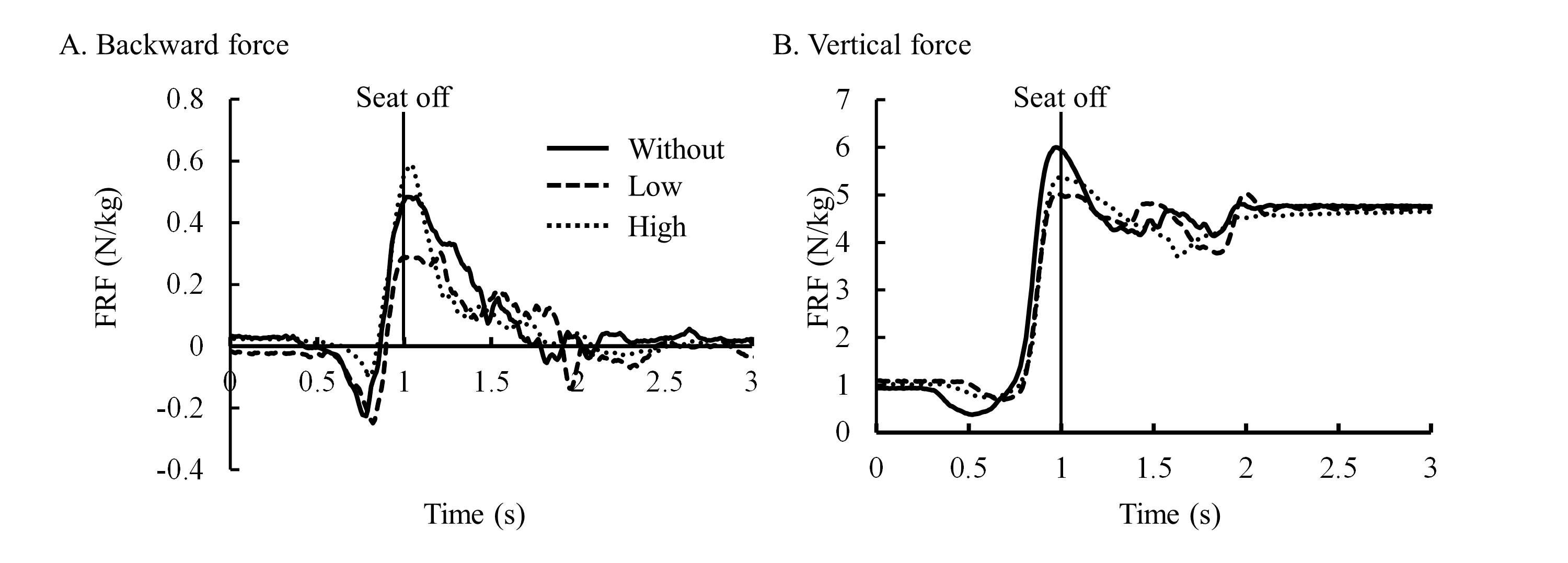

Supplement: S3 Fig — Vertical solid line indicates the seat-off. (TIF) [file pone.0133747.s003.tif]
